# C-Ring Structure-Dependent Redox Properties of Flavonoids Regulate the Expression of Bioactivity

**DOI:** 10.3390/antiox15020194

**Published:** 2026-02-02

**Authors:** Taiki Fushimi, Kenta Aso, Takafumi Shimizu, Chie Hirahata, Kento Hiroki, Daichi Shinmura, Yasuyuki Fujii, Mitsugu Akagawa, Ali S. Abdelhameed, Vittorio Calabrese, Shu Taira, Yoshitomo Suhara, Naomi Osakabe

**Affiliations:** 1 Department of Food and Nutrition, Institute of Biomedical Sciences, Tokushima University Graduate School, Tokushima 770-8503, Japan; fushimi.taiki@tokushima-u.ac.jp (T.F.); akagawa@tokushima-u.ac.jp (M.A.); 2 Systems Engineering and Science, Graduate School of Engineering and Science, Shibaura Institute of Technology, Saitama 337-8570, Japan; suhara@sic.shibaura-it.ac.jp; 3 Central Research Institute, ITO EN, Ltd., Tokyo 151-8550, Japan; k-aso@itoen.co.jp; 4 Department of Bioscience and Engineering, Faculty of System Science and Engineering, Shibaura Institute of Technology, Saitama 337-857, Japan; bn20003@shibaura-it.ac.jp (T.S.);; 5 SIT Laboratory, Shibaura Institute of Technology, Saitama 337-857, Japan; fujii.yasuyuki.x1@shibaura-it.ac.jp; 6 Department of Pharmaceutical Chemistry, College of Pharmacy, King Saud University, Riyadh 11451, Saudi Arabia; asaber@ksu.edu.sa; 7 Department of Biomedical and Biotechnological Sciences, University of Catania, 95124 Catania, Italy; vittorio.calabrese@unict.it; 8 Faculty of Food and Agricultural Sciences, Fukushima University, Fukushima 960-1248, Japan; staira@agri.fukushima-u.ac.jp

**Keywords:** flavonoid, C-ring, stability, reactive oxygen species, sympathetic nerve

## Abstract

(−)-Epicatechin (EC), taxifolin (Tax), and quercetin (Q) are flavonoids with different C-ring structures. We compared their physicochemical properties and biological activities. The comparison of their stability and redox properties was conducted at mildly acidic or neutral conditions mimicking the plant vacuole or gut, and their sympathetic hyperactivation ability was examined using catecholamine (CA) excretion and blood flow. At pH 5, flavonoids were stable, but at pH 7, their retention rates decreased in the order EC > Q > Tax. LC-MS analysis detected brown oxidized oligomers in EC, while Tax and Q primarily yielded degradation products. All flavonoids exhibited O_2_^•−^ scavenging activity after incubation at pH 5. At pH 7 after 45-min, EC and Tax promoted O_2_^•−^, while Q only scavenged O_2_^•−^ after 24-hr incubation, EC’s properties decreased but Tax’s properties enhanced. Computational chemistry analysis indicated EC has higher reactivity compared to Tax and Q. EC caused a significant increase in CA excretion and blood flow, which were not observed with Tax or Q or 24-h cultured EC. These results suggest that the C-ring structure of flavonoids plays a crucial role in their stability and redox properties. Furthermore, reactive oxygen species generated by flavonoids in the gut may exert beneficial effects through sympathetic activation.

## 1. Introduction

Flavonoids represent a group of plant secondary metabolites characterized by a diphenylpropane (C6-C3-C6) skeleton and two benzene rings (A and B rings) [1]. It is well established that the flavonoid is produced in a series of cytoplasmic (pH 7.3–8.0), apoplastic (pH 4.0–6.3), or vacuolar lumen (pH 5.0–6.0) environments [2,3]. The redox properties of flavonoids are dependent upon their chemical structure. It is presumed that these properties are affected by the behavior, solubility, and polarity of the flavonoids in aqueous solution, particularly regarding pH [4,5]. Flavonoids are subjected to various pH conditions following ingestion as they traverse the gastrointestinal tract. These conditions, characteristic of the stomach, small intestine, and duodenum, span a wide range, from highly acidic to slightly alkaline [6]. The stomach exhibits a relatively high acidity level, with a pH range of 1.0 to 2.0, reaching an average of 6.1 in the duodenum, 7.1 in the middle small intestine, and 7.5 in the distal small intestine. It has been established that the pH level undergoes a transient decline to approximately 6.0 near the cecum. Thereafter, it exhibits an ascent towards the rectum, ultimately attaining about 7.0 in the region of the large intestine’s exit [7]. The duration of transit through the digestive tract varies according to the type of food and its constituent ingredients; however, it has been reported that the following times are typical: a few minutes in the oral cavity and esophagus, 1 to 3 h in the stomach, 2 to 6 h in the small intestine, and more than 24 h in the large intestine [8].

A minor proportion of ingested flavonoids is absorbed from the small intestine and metabolized into the bloodstream; however, most of them are transported unaltered to the lower part of the digestive tract. It has been documented that some of these are metabolized by intestinal bacteria, resulting in the production of low molecular weight metabolites that are subsequently absorbed [9,10,11]. Nevertheless, in consideration of the electrochemical characteristics of polyphenols, oxidation and related decomposition may have already occurred in regions with a neutral pH, such as oral cavity and the small intestine.

Conversely, our previous studies have shown that a single oral intake of flavanol EC leads to significant changes in the cardiovascular and metabolic systems through increased sympathetic nervous activity [12,13]. For instance, numerous intervention studies have shown that regularly consuming flavanols such as EC can lower blood pressure [14,15,16]. Furthermore, it is well known that consuming 200 mg or more of flavanols in a single intake increases the flow-mediated dilatation (FMD) value [17,18,19]. However, while only a small percentage of the flavanol EC is absorbed, it is metabolized in intestinal epithelial cells and converted into water-soluble conjugates [20,21]. This means that the original form of epicatechin is barely present in the bloodstream [12,22]. Consequently, the mechanism behind this improvement in vascular endothelial function was unclear for a long time. Our previous studies using experimental animals demonstrated a reduction in blood pressure following repeated flavanol administration and a transient increase in blood pressure, heart rate, and skeletal muscle blood flow. There was also an increase in eNOS expression in the vascular endothelium after a single dose. Furthermore, these effects were cancelled by adrenergic receptor antagonists, indicating that flavanol administration enhances sympathetic nerve activity [23,24]. The dose of EC that produced increased FMD values in the intervention trial was consistent with the dose that increased skeletal muscle blood flow in animal studies; we also used this dose in the present study. These symptoms resemble the changes in hemodynamics observed during exercise due to increased sympathetic activity. Specifically, transient increases in blood flow led to increased eNOS expression and vascular relaxation (as evidenced by elevated FMD values), and repeated administration induced angiogenesis via vascular endothelial growth factor secreted after evoking shear stress, resulting in a decrease in blood pressure [12]. The increase in sympathetic nerve activity induced by flavanols is also demonstrated by elevated blood adrenaline and noradrenaline levels observed 2–4 h after a single dose [25]. It has been suggested that the physiological changes caused by administration of these flavanol ECs are due to the induction of a stress response [26,27]. Therefore, in this experiment, we decided to use two indicators of increased sympathetic nervous system activity arising from stress response reactions: urinary catecholamine concentration—secreted into the bloodstream from sympathetic nerve endings or the adrenal glands and excreted in urine—and changes in skeletal muscle blood flow caused by transient increases in blood pressure and heart rate.

**Figure 1:**
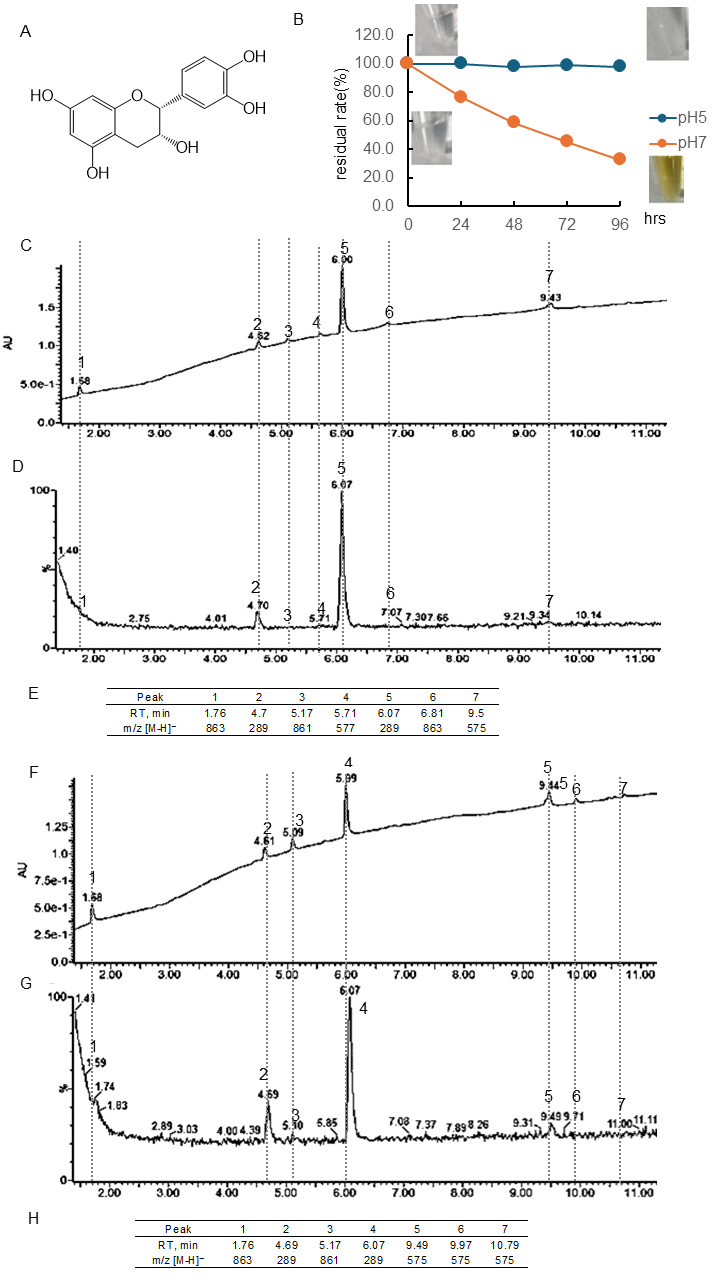
Stability of (−)-epicatechin (EC) under different pH conditions. (**A**) Structural formula of EC; (**B**) EC residual rate and color change over time at pH 5 or 7. Data represent the average of three independent experiments. (**C**) Chromatogram of EC incubated at pH 7 for 48 h (detected photodiode array, PDA); (**D**) chromatogram of EC incubated at pH 7 for 48 h (detected MS); (**E**) retention time of the peak and obtained in (**D**) and *m*/*z* [M-H]^−^; (**F**) chromatogram of EC incubated at pH 7 for 96 h (detected PDA); (**G**) chromatogram of EC incubated at pH 7 for 96 h (detected MS); (**H**) retention time of the peak and obtained in (**G**) and *m*/*z* [M-H]^−^.

**Figure 2:**
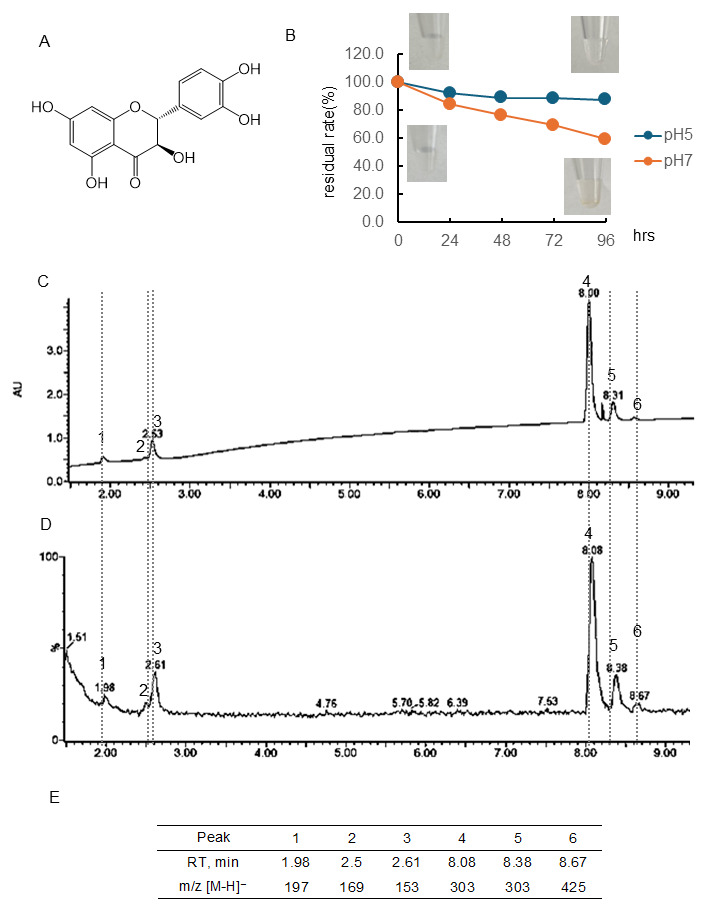
Stability of taxifolin (Tax) under different pH conditions. (**A**) Structural formula of Tax; (**B**) Tax residual rate and color change over time at pH 5 or 7. Data represent the average of three independent experiments; (**C**) chromatogram of Tax incubated at pH 7 for 96 h (detected photodiode array, PDA); (**D**) chromatogram of Tax incubated at pH 7 for 96 h (detected MS); (**E**) retention time of the peak and obtained in (**D**) and *m*/*z* [M-H]^−^.

**Figure 3:**
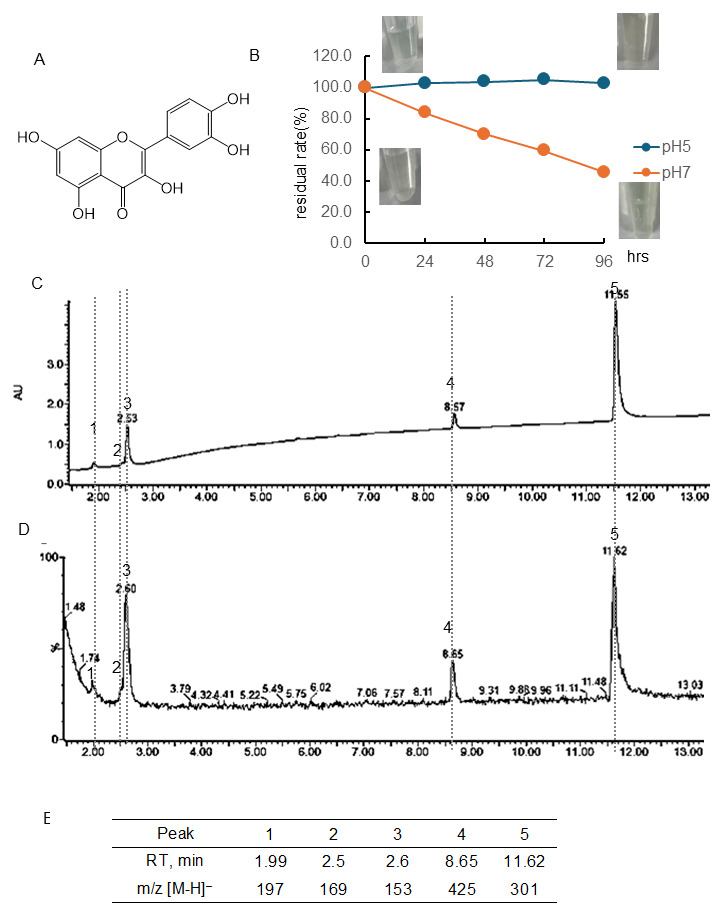
Stability of quercetin (Q) under different pH conditions. (**A**) Structural formula of Q; (**B**) Q residual rate and color change over time at pH 5 or 7. Data represent the average of three independent experiments; (**C**) chromatogram of Q incubated at pH 7 for 96 h (detected photodiode array, PDA); (**D**) chromatogram of Q incubated at pH 7 for 96 h (detected MS); (**E**) retention time of the peak and obtained in (**D**) and *m*/*z* [M-H]^−^.

Accordingly, the present study compared the stability, redox properties, and sympathetic neuron activation ability of three flavonoids with distinct C-ring structures: (−)-epicatechin (EC, Figure 1A), a flavanol; taxifolin (Tax, Figure 2A), a flavanonol; and quercetin (Q, Figure 3A), a flavonol. These flavonoids have identical A-ring and B-ring, but different C-ring structures. Q is known as a flavonoid widely found in fruits and vegetables, possessing potent antioxidant properties. Numerous health benefits have been reported, including anti-inflammatory effects, anti-cancer activity, and cardiovascular protection [28,29]. Recent studies have also found that when combined with tyrosine kinase inhibitors, it exhibits senolytic effects, removing senescent cells [30,31]. Tax, known as dihydroquercetin, belongs to the flavonol group and is abundant in conifers such as *Siberian larch* [32]. Recent studies report that taxifolin, like quercetin, possesses strong antioxidant and anti-inflammatory effects and holds potential for preventing various lifestyle-related diseases [33,34]. EC is found in tea, cacao, grapes, and other sources, with reported health benefits spanning vascular function, muscle metabolism, and anti-aging effects [35]. Tax is synthesized through the phenylpropanoid pathway and the flavonoid biosynthetic pathway in plants. Q is produced by flavonol synthase (FLS, EC 1.14.20.6) from Tax (dihydroquercetin). EC is also produced from Tax through the consecutive actions of dihydroflavonol 4-reductase (DFR, EC 1.1.1.219), anthocyanin synthase (ANS, also known as flavonoid 3′,5′-hydroxylase, EC 1.14.11.12), and anthocyanidin reductase (ANR, EC 1.3.1.77) [36,37,38].

In the study of their stability and reactivity with O_2_^•−^, the changes were observed under conditions of pH 5.0, which is like the pH at which flavonoids exist in plant bodies, and pH 7.0, which is similar to the pH of the oral cavity and intestinal tract, and they were compared. Furthermore, to ascertain the relationship between the chemical structures and the stability and redox properties of the compounds in question, molecular reactivity was calculated using computational science techniques. Moreover, to elucidate the relationship between these physicochemical differences and their bioactive properties, particularly regarding sympathetic nerve activation, we examined the effects on urinary catecholamine (CA) concentrations and skeletal muscle arteriolar blood flow using an experimental animal model.

## 2. Materials and Methods

### 2.1. Materials

EC (E1226) and quercetin (P0042) were purchased from Tokyo Chemical Industry Co., Ltd. (Tokyo, Japan). (+)-trans-taxifolin (≧99%) was purchased from Nagara Science Co., Ltd. (NS510102, Tokyo, Japan). The production of O_2_^•−^ was quantified using the MPEC (AB-2950), xanthine (X, 241–00013), and xanthine oxidase (XOD, XTO-212) as previously reported. Sodium carbonate (199-01585) and gallic acid monohydrate (077-06092) were purchased from Fujifilm Wako Pure Chemical Corporation (Tokyo, Japan).

### 2.2. Animals and Diets

Male Wistar rats (7–8 weeks old) were obtained from Saitama Experimental Animal Supply (Tokyo, Japan). Ten-week-old male C57BL/6J mice were obtained from CLEA Japan, Inc. (Tokyo, Japan). The breeding conditions, such as the cages and the number of animals in each cage, were carried out in accordance with the ARRIVE guidelines. The protocol was approved by the Animal Experimentation Committee of the Shibaura Institute of Technology (approval number AEA23008), and the animals were raised humanely according to the guidelines. The animals were housed at a room temperature of 24–26 °C under a 12-h light/dark cycle (light cycle: 7:00–19:00; dark cycle: 19:00–7:00) and had free access to food and water. The solid diet (MF) for laboratory animals was obtained from Oriental Yeast Co., Ltd. (Tokyo, Japan). During the two-week acclimation period, the animals were allowed to get used to their new habitat and reverse light-dark cycle. To reduce anxiety behaviors, a trained handler wearing mouse-scented gloves handled each mouse for one minute between 10:30 and 11:00 a.m. During this handling, the mouse was captured in an open or cupped hand without physical restraint. All studies were conducted with animals that did not exhibit agonistic behaviors (e.g., chasing, lunging, biting, fleeing) throughout the experimental period, in order to avoid the effects of social dominance in animal groups.

### 2.3. Stability of Flavonoids Under Different pH Conditions

Each flavonoid was dissolved at a concentration of 2 mM in 0.01 M phosphate buffer at pH 2.8. The solution was dissolved at a concentration of 200 μM in 0.1 M phosphate buffer at pH 5.0 or pH 7.0, which is a model for the vacuole of plants or the oral cavity and small intestine. It was sampled before incubation and after 24, 48, 72, and 96 hrs. After incubation, the samples were diluted 10-fold with 0.01M pH 2.8 phosphate buffer to a final concentration of 20 μM. Samples were analyzed with an ACQUITY Premier UPLC system (Nihon Waters K.K., Tokyo, Japan) equipped with a pump, a degasser, an autosampler, and a photodiode array detector (PDA). Samples (5 μL) were injected into an ACQUITY Premier BEH C18 column (130Å, 1.7 µm, 2.1 mm × 100 mm, Waters). The flow rate was 0.3 mL/min and the column temperature was 40 °C. The mobile phases were water (A), acetonitrile (B), and 1% (*v*/*v*) formic acid (C) and elution was performed using a linear gradient with C fixed at 10%: 5% B from 0.0 to 1.0 min, 5% to 45% B from 1.0 to 15.0 min, 45% to 90% from 15.0 to 15.1 min, 90% B from 15.1 to 19 min 90% to 5% from 19.0 to 19.1 min, and 5% B from 19.1 to 24.0 min. The range of detection wavelengths was set to 230−470 nm, and data were recorded at 20 points/sec. Then, samples were analyzed with Xevo TQ-S micro (Nihon Waters K.K., Tokyo, Japan) using an electrospray ionization source operated in positive and negative mode. Data were collected from 150 to 1500 *m*/*z* for full scan. Other MS parameters are as follows: capillary voltage was 1 kV; source temperature was 150 °C; desolvation temperature was 500 °C; desolvation gas flow was 1000 L/hr; and cone gas flow was 50 L/hr. The residual amount of each flavonoid was calculated using the peaks of chromatography using PDA.

### 2.4. Redox Property of Flavonoids Under Different pH Conditions

To assess the redox characteristics of each flavonoid, the experiment was conducted using the method by Fushimi et al. [39]. MPEC is a *Cypridina* luciferin analog developed as an imidazopyrazinone-based probe with oxygen-selective properties. A dilution series of test chemicals was prepared using 0.01 M phosphate buffer (pH 2.8) to ensure the stability of polyphenols such as EC, Tax, and Q under acidic conditions. The test chemicals were incubated at 37 °C in 0.1 M phosphate buffer (pH 5.0 or 7.0). Considering the time required for test chemicals to reach or be eliminated from the intestine following oral ingestion or to be approximately emptied from the digestive tract, the incubation period was set at 45 min or 24 h. The chemiluminescence of the reaction solution containing the test chemical dilution series (7.85 µM MPEC and 150 mU/mL XOD) was then measured promptly following the addition of 30 µM X.

### 2.5. Method of Computational Analysis of the Reactivity of Flavonoid

#### 2.5.1. Calculation of a Single Molecular Dynamics Simulation

Single molecular dynamics simulations were performed to study the molecular fluctuations in water solvents. These analyses were performed using Molecular Operating Environment (MOE, ver. 2022.02). The analysis began with the construction of a simulation box measuring 40 Å on each side (40 Å × 40 Å × 40 Å), in which each flavonoid molecule (EC, Tax, and Q) was placed in the center. Energy minimization was performed on the entire system to minimize the total potential energy and ensure that the starting point of the simulation is stable. Then the entire system was then heated up to 300 K over 100 ps, and equilibrated under constant Number of particles, Volume, and Temperature (NVT) and constant Number of particles, Pressure, and Temperature (NPT) conditions with durations of 100 ps and 200 ps and step sizes of 50,000 and 100,000 steps, respectively. Finally, 50,000 ps simulations were performed, and the root-mean-square fluctuation (RMSF) of the atomic positions of each flavonoid were analyzed, alongside the root-mean-square deviation (RMSD) to evaluate the structural stability and deviations of the flavonoid conformations throughout the simulations.

#### 2.5.2. Calculation of the Angle and Dihedral Angle

The angles and dihedral angles were calculated using DFT-optimized calculations. All compound calculations were performed using the Gaussian software package. Molecular visualization was performed using Winmostar (V11.7.6, X-Ability Co., Ltd., Tokyo, Japan, 2024). The DFT method was used to optimize the molecular structures using the B3LYP-D3 hybrid functional at the 6-311++G (d, p) basis set. To verify that each optimized structure represents a global minimum, the frequencies of the structures were estimated by the IR method. The solvent effect of different polarities (water: ε = 78.36) was done with the solvation model based on density (SMD) implemented in the Gaussian software.

### 2.6. Method of Quantitative Determination of Urinary Catecholamine Concentrations After Administration of Flavonoid

It has been suggested that brief periods of social isolation in metabolic cages can markedly alter sympathetic nervous system activity in mice [40]. Our preceding research demonstrated that co-housing two mice in metabolic cages markedly reduced the stress response associated with single housing [41]. Consequently, in the present study, we employed this approach to examine flavonoids’ sympathetic nervous system activity. Following a 48-h acclimation period, urine was collected for 24 h using a tube containing 20 µL of 2.5 mol/L HCl following oral administration of 100 µg/kg test chemicals. We performed a dose-response study of EC on urinary catecholamine excretion and found that a significant increase in adrenaline excretion was observed at 100 μg/kg, so we selected this dose. The data from the dose-response study are shown in the Supplementary File (Figure S1).

### 2.7. Measurement Method of Cremaster Arteriolar Blood Flow After Administration of Flavonoid

Thirty rats were divided into five treatments as following groups; vehicle (4 mL/kg in 3% Tween80 solution), 10 µg/kg EC, Tax, Q, or after 24 h incubation EC (10 µg/kg; n = 6 each). Measurement of BF was performed according to a previously reported method [39]. Briefly, rats were anesthetized with urethane (1 g/kg s.c.), and a gastric tube was inserted into the stomach. Each chemical was infused at a 1.0 mL/min rate to avoid a circulatory reflex that this procedure can induce. The cremaster muscle was exteriorized, and the surface was perfused with phosphate-buffered saline. We performed a dose-response study of EC on blood flow and found that a significant increase was observed at 10 μg/kg, so we selected this dose. The data from the dose-response study are shown in the Supplementary File (Figure S2). After a post-surgical equilibration period, baseline measurements of cremaster arteriole blood flow were conducted for 10 min. Each treatment was orally administered to animals through the gastric tube. Blood flow in the cremaster artery was monitored for 60 min before and after compound administration using a laser Doppler blood flow meter (Periscan PIM-2, Perimed Co., Ltd., Stockholm, Sweden).

### 2.8. Data Analysis and Statistical Methods

The sample size was determined from the preliminary experiment results using a power test with a significance level of 0.05 and power 0.9. All quantitative assessments were carried out blindly. The normal distribution of the sample was tested using the Shapiro–Wilk test. No data was excluded from the analysis. All data were expressed as mean ± standard deviation. Statistical analysis was performed in Prism 10 (Version 10.2.3, GraphPad Software) and performed using one or two-way analysis of variance or Kruskal–Wallis test and post hoc comparisons using Dunnett’s test or Dunn’s tests. The significance levels were defined at # *p* < 0.1, * *p* < 0.05, ** *p* < 0.01, *** *p* < 0.001, **** *p* < 0.0001.

## 3. Results

### 3.1. Stability of Flavonoids at Different pH Conditions

#### 3.1.1. Stability of (−)-Epicatechin(EC)

Figure 1B and Table S1 showed the quantitative change in EC concentration over 96 h at pH 5 and 7 and the photo of the sample. The residual rate was calculated using the peaks of chromatography using PDA. The chromatogram of EC following 48 or 96 h of incubation at pH 7 using a PDA detector was displayed in Figure 1C or D, and the chromatogram employing an MS detector was presented in Figure 1F or G. The retention time (RT) and [M-H]− ion at *m*/*z* of the peaks obtained were shown in Figure 1E or H. At pH 5, there was almost no change in concentration, but at pH 7, the concentration decreased over time, i.e., the residual rate was at 24 h, 76.4%; 48 h, 58.4%; 72 h, 45.3%; 96 h, 32.6%, respectively. Immediately after dissolution, the EC was colorless, but after 96 h, noticeable browning was observed. The parent compound EC had a peak at 6.07 min, and the isomer (+)-catechin had a peak at 4.7 min. Following a 48- and 96-hr incubation period, it was observed that common peaks appeared at 1.76 min ([M-H]− ion at *m*/*z*, 863) and 5.17 min ([M-H]− ion at *m*/*z* -, 861). After 48 h of incubation, peaks were detected at 5.71 min ([M-H]− ion at *m*/*z*, 577), 6.81 min ([M-H]− ion at *m*/*z*, 863), and 9.5 min ([M-H]− ion at *m*/*z*-, 575). However, these peaks were not observed after 96 h of incubation, and peaks with [M-H]− ion at *m*/*z* 575 were observed at 9.49, 9.97, and 10.79 min.

#### 3.1.2. Stability of (+)-Taxifolin (Tax)

Figure 2B and Table S1 showed the quantitative change in Tax concentration over 96 h at pH 5 and 7 and the color change. Tax following 96 h of incubation at pH 7 using a PDA detector was displayed in Figure 2C, and the chromatogram employing an MS detector was presented in Figure 2D. The chromatogram of RT and [M-H]− ion at *m*/*z* of the peaks obtained are shown in Figure 2E. At a pH of 5, the residual rate of Tax was approximately 90% after 24 h of incubation, and this value remained almost constant thereafter. At pH 7, the concentration decreased over time, i.e., the residual rate was at 24 h, 84.4%; 48 h, 76.4%; 72 h, 69.4%; 96 h, 59.3%, respectively. The residual rate of Tax at pH 7 was markedly higher than that of EC. As shown in the photo (Figure 2B), no color change was observed in the Tax solution before and after 96 h of incubation. The parent compound Tax had a peak at 8.08 min, and one of the isomers had a peak at 8.38 min ([M-H]− ion at *m*/*z*, 303). Following a 96-hr incubation period, it was observed that major peaks appeared at 1.98 min ([M-H]− ion at *m*/*z*, 197), 2.50 min ([M-H]− ion at *m*/*z*-, 169), 2.61 min ([M-H]− ion at *m*/*z*, 153), and 8.67 ([M-H]− ion at *m*/*z*, 425).

#### 3.1.3. Stability of Quercetin (Q)

Figure 3B and Table S1 showed the quantitative change in Q concentration over 96 h at pH 5 and 7 and the color change. The chromatogram of Q following 96 h of incubation at pH 7 using a PDA detector was displayed in Figure 3C, and the chromatogram employing an MS detector was presented in Figure 3D. The RT and [M-H]^−^ ion at *m*/*z* of the peaks obtained are shown in Figure 3E. At pH 5, there was almost no change in concentration. At pH 7, the concentration decreased over time, i.e., the residual rate was at 24 h, 83.7%; 48 h, 69.9%; 72 h, 59.5%; 96 h, 45.6%, respectively. The present study found that the residual rate of Q at a pH of 7 was higher than the rates of EC and lower than that of Tax. As shown in the photo (Figure 3B), no color change was observed in the Q solution before and after 96 h of incubation. The parent compound Q had a peak at 11.62 min. Following a 96-hr incubation period, it was observed that major peaks appeared at 1.99 min ([M-H]^−^ ion at *m*/*z*, 197), 2.50 min ([M-H]^−^ ion at *m*/*z*, 169), 2.60 min ([M-H]^−^ ion at *m*/*z*, 153), and 8.65 ([M-H]^−^ ion at *m*/*z*, 425). The RT and [M-H]^−^ ion at *m*/*z* values of these four peaks were consistent with those obtained with Tax.

### 3.2. Redox Property of Flavonoids

#### 3.2.1. Redox Property of (−)-Epicatechin (EC)

**Figure 4:**
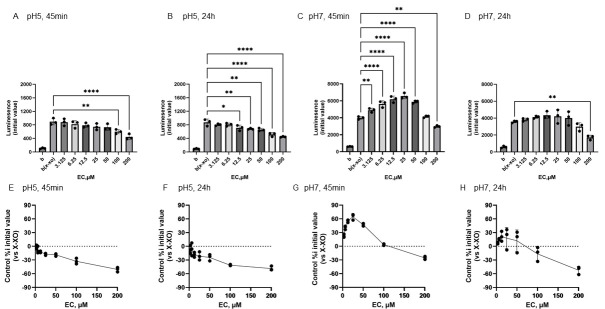
Redox properties of (−)-epicatechin (EC) at 45 min or 24 h at different pH. The upper panel displays the average initial chemiluminescence at each concentration, while the lower panel shows the relative luminescence intensity compared to the control (without flavonoids). The two graphs on the left show the results after incubation at pH 5 for 45 min (**A**,**E**) or 24 h (**B**,**F**). The two graphs on the right show the results after incubation at pH 7 for 45 min (**C**,**G**) or 24 h (**D**,**H**). Data represents the mean ± SD of three independent experiments, * *p* < 0.05, ** *p* < 0.01,, **** *p* < 0.0001 compared to the positive control (X-XOD), after one-way ANOVA followed by Dunnett’s test.

Figure 4 shows the reactivity of EC toward O_2_^•−^ generated by X-XOD. The upper panel displays the average initial chemiluminescence at each concentration, while the lower panel shows the relative luminescence intensity compared to the control (without flavonoids). From left to right, the results are shown after incubation at pH 5 for 45 min (Figure 4A,E) or 24 h (Figure 4B,F), and incubation at pH 7 for 45 min (Figure 4C,G) or 24 h (Figure 4D,H). At pH 5, the mean initial chemiluminescence decreased in a concentration-dependent manner upon EC addition at both 45 min and 24 h post-incubation (Figure 4A,B,E,F). The mean initial chemiluminescence was significantly reduced compared to the control at 100–200 µM EC after 45 min incubation and at 12.5–200 µM EC after 24 h incubation. The maximum reduction in initial luminescence was similar at 45 min and 24 h incubation, decreasing by approximately 50% at 200 µM (Figure 4F,H). At pH 7.0, luminescence increased markedly at 45 min post-incubation in the low EC groups (Figure 4C,G), but these changes were not observed at 24 h post-incubation (Figure 4D,H). The mean initial chemiluminescence increased compared to the control at 3.125–50 µM, peaking at 25 µM, but did not increase at 24 h post-incubation. At 200 µM, a decrease in luminescence was observed at both 45 min and 24 h post-incubation. The increase in luminescence intensity at 45 min was dose-dependent, peaking at approximately 64.5% increase at 25 µM, whereas at 200 µM, luminescence intensity decreased by approximately 25.4% (Figure 4G). After 24-h incubation, no increase in luminescence intensity was observed in EC, and a decrease of approximately −52.2% was observed at 200 µM (Figure 4H).

#### 3.2.2. Redox Property of Taxifolin (Tax)

**Figure 5:**
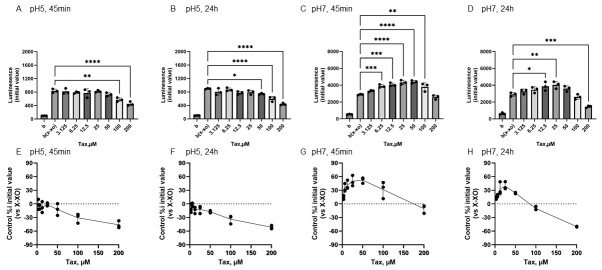
Redox properties of taxifolin (Tax) at 45 min or 24 h at different pH. The upper panel displays the average initial chemiluminescence at each concentration, while the lower panel shows the relative luminescence intensity compared to the control (without flavonoids). The two graphs on the left show the results after incubation at pH 5 for 45 min (**A**,**E**) or 24 h (**B**,**F**). The two graphs on the right show the results after incubation at pH 7 for 45 min (**C**,**G**) or 24 h (**D**,**H**). Data represents the mean ± SD of three independent experiments, * *p* < 0.05, ** *p* < 0.01, *** *p* < 0.001, **** *p* < 0.0001 compared to the positive control (X-XOD), after one-way ANOVA followed by Dunnett’s test.

Figure 5 shows the reactivity of Tax toward O_2_^•−^ generated by X-XOD. The upper panel displays the average initial chemiluminescence at each concentration, while the lower panel shows the relative luminescence intensity compared to the control (without flavonoids). From left to right, the results are shown after incubation at pH 5 for 45 min (Figure 5A,E) or 24 h (Figure 5B,F), and incubation at pH 7 for 45 min (Figure 5C,G) or 24 h (Figure 5D,H).

At pH 5, Tax addition caused a concentration-dependent decrease in the mean initial chemiluminescence at both 45 min and 24 h post-incubation (Figure 5A,B). The mean initial chemiluminescence was significantly reduced by Tax addition at concentrations of 100–200 µM after 45 min of incubation (Figure 5A) and significantly reduced compared to the control group at concentrations of 50–200 µM after 24 h of incubation (Figure 5B). The maximum reduction in initial luminescence was similar at 45 min and 24 h post-incubation, decreasing by approximately 50% at 200 µM (Figure 5E,F). At pH 7.0, luminescence increased in the low Tax concentration group after 45 min incubation, and these changes were also observed after 24 h incubation (Figure 5C,D). The mean initial chemiluminescence peaked at 50 µM after 45 min of incubation and increased from 6.25 µM to 100 µM (Figure 5G). After 24 h of incubation, similar enhancement was observed at 12.5 µM and 50 µM, while a decrease in luminescence was observed at 200 µM (Figure 5H). The increase in luminescence intensity after 45 min of incubation was dose-dependent, peaking at approximately 52.4% increase at 50 µM (Figure 5G). The increase in luminescence intensity after 24 h of incubation was also dose-dependent, showing approximately 39.2% increase at 25 µM. Conversely, a decrease of approximately 50.0% was observed at 200 µM (Figure 5H).

#### 3.2.3. Redox Property of Quercetin (Q)

**Figure 6:**
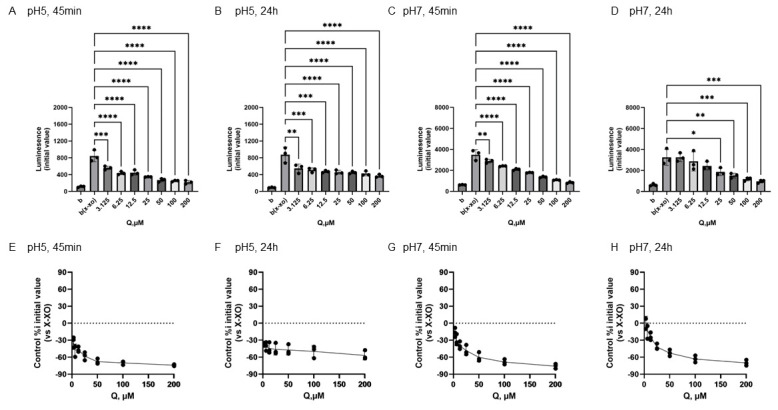
Redox properties of quercetin (Q) at 45 min or 24 h at different pH. The upper panel displays the average initial chemiluminescence at each concentration, while the lower panel shows the relative luminescence intensity compared to the control (without flavonoids). The two graphs on the left show the results after incubation at pH 5 for 45 min (**A**,**E**) or 24 h (**B**,**F**). The two graphs on the right show the results after incubation at pH 7 for 45 min (**C**,**G**) or 24 h (**D**,**H**). Data represents the mean ± SD of three independent experiments, * *p* < 0.05, ** *p* < 0.01, *** *p* < 0.001, **** *p* < 0.0001 compared to the positive control (X-XOD), after one-way ANOVA followed by Dunnett’s test.

In Figure 6, the results of Q to O_2_^•−^ produced by X-XOD. The upper panel displays the average initial chemiluminescence at each concentration, while the lower panel shows the relative luminescence intensity compared to the control (without flavonoids). From left to right, the results are shown after incubation at pH 5 for 45 min (Figure 6A,E) or 24 h (Figure 6B,F), and incubation at pH 7 for 45 min (Figure 6C,G) or 24 h (Figure 6D,H).

The mean initial chemiluminescence decreased in a concentration-dependent manner with the addition of Q at all of the experiments (Figure 6A–D). The average of the initial chemiluminescence was significantly reduced by Q at concentrations from 25 to 200 µM 24 h after incubation at pH 7 (Figure 6D), but the similar decrease in light emission intensity was recognized by the addition of Q of 3.125 μM or more under the other three conditions (Figure 6A–C). The reducing ratio was from −76.1% and −70.6%, respectively, at the maximum concentration of 200 µM (Figure 6C–F).

### 3.3. Computational Analysis of the Reactivity of Flavonoids

Since significant pH-dependent differences were observed in the stability and reactivity with O_2_^•−^ of EC, Tax, and Q, we decided to computationally clarify the properties of each molecule.

#### 3.3.1. Single Molecular Dynamics Simulations

**Figure 7:**
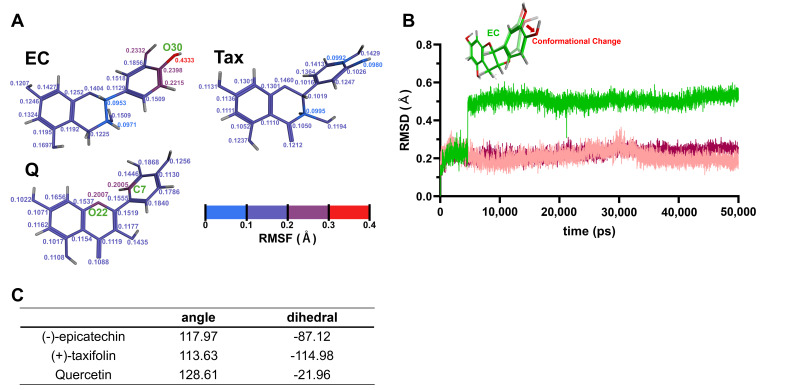
Single molecular dynamics simulations of EC, Tax, Q. (**A**), RMSF values calculated from the atomic positions of each flavonoid during the production run. Higher RMSF values correspond to regions with greater molecular fluctuations, with the color transitioning from blue to red as the RMSF values increase. Notably, the B-ring of EC shows the highest fluctuation, particularly at the oxygen atom at the 4′ position. (**B**) Time evolution of RMSD values during the production run. The 0 ps point represents the initial structure after NVT and NPT equilibrations. A marked structural change in the B-ring of EC was observed between 4600 ps and 4610 ps, while the RMSD values for Tax and Q remained stable throughout the run. (**C**) Calculation results of the angle and dihedral angle.

The results of the single molecular dynamics simulations for each flavonoid are shown in Figure 7. Figure 7A depicts RMSF values calculated from the atomic positions of each flavonoid saved during the 50,000 ps production run. Regions with larger atomic positional variations, i.e., greater molecular fluctuations, correspond to higher RMSF values. In Figure 7A, the color transitions from blue to red as the RMSF values increase. Notably, the RMSF values were highest in the B-ring of EC, with the oxygen atom at the 4′ position (O30) showing the greatest fluctuation (0.4333 Å). In the case of Tax, the RMSF values were generally lower. For Q, relatively big fluctuations were observed at the carbon atom at the 2′ position of the B-ring (C7) and the oxygen atom in the C-ring (O22), both showing almost the same RMSF values (0.2005 and 0.2007 Å, respectively). Figure 7B illustrates the time evolution of root-mean-square deviation (RMSD) values during the 50,000 ps production run. The 0 ps point represents the initial structure of the atoms after Number of particles, Volume, and Temperature (NVT) and Number of particles, Pressure, and Temperature (NPT) equilibrations. Larger RMSD values indicate greater structural deviations from this initial structure. Notably, the significant structural change in the B-ring of EC was observed between 4600 ps and 4610 ps. In contrast, the RMSD values for Tax and Q remained relatively stable throughout the production run. The atomic numbers of all atoms in EC, Tax, and Q, along with the detailed atomic parameters employed during the simulations and the corresponding RMSF values, are provided in Tables S1–S3.

#### 3.3.2. Calculation of the Angle and Dihedral Angle

The angle (°) around the C3-C2-C1′ bond was 117.97° in EC, 113.63° in Tax, and 128.61° in Q (Figure 7C). The dihedral angle (τ) around the C2-C1′ bond was −87.12τ in EC, −114.18τ in Tax, and −21.96τ in Q (Figure 7C).

### 3.4. Quantitative Determination of Urinary Catecholamine Concentrations After Administration of Flavonoids

**Figure 8:**
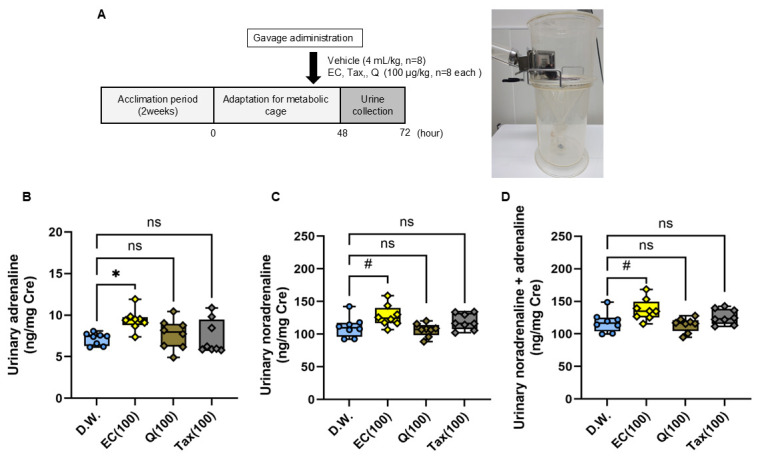
Twenty-four-hour urinary catecholamine excretion after a single oral dose of each flavonoid. (**A**) experimental procedure; (**B**) adrenaline (AD); (**C**) noradrenalin (NA); (**D**) total catecholamine (CA). The excretion of CA was expressed as a ratio with the urinary creatinine concentration. Data represents the mean ± SD (n = 8, each), ns: not significant, # *p* < 0.1, * *p* < 0.05 compared to vehicle, after Kruskal–Wallis test followed by followed by Dunn’s tests.

The excretion of catecholamines in urine over 24 h following a single oral administration of each flavonoid is illustrated in Figure 8. Urinary adrenaline excretion was found to be significantly increased in the EC group in comparison to the vehicle group; however, no difference was observed between the Tax or Q groups (Figure 8B). Similarly, urinary noradrenaline excretion showed a tendency to increase in the EC group in comparison to the vehicle group, yet no difference was detected between the Tax or Q groups (Figure 8C). Finally, total catecholamine excretion showed a tendency to increase in the EC group compared to the vehicle group, but no difference was observed between the Tax or Q groups (Figure 8D).

### 3.5. Measurement of Cremaster Arteriolar Blood Flow After Administration of Flavonoids

**Figure 9:**
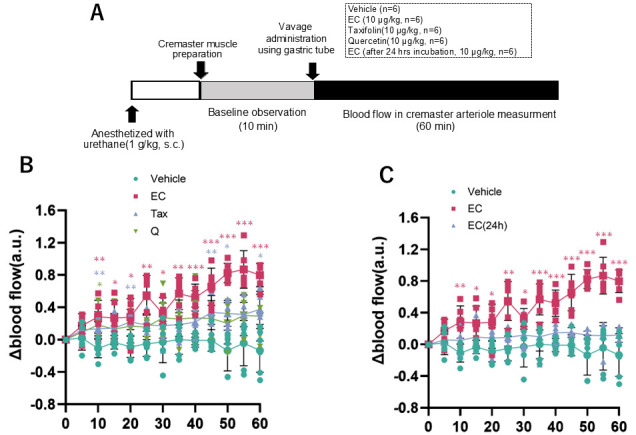
Blood flow in rat cremaster arteriole after a single oral dose of each flavonoid. (**A**), experimental procedure; (**B**) comparison of vehicle, 10 µg/kg EC, 10 µg/kg Tax, and 10 µg/kg Q; (**C**) comparison of vehicle, EC, and 24-h after incubation of EC. Data represents the mean ± SD (n = 6, each), * *p* < 0.05, ** *p* < 0.01, *** *p* < 0.001, compared to vehicle, after two-way ANOVA tests followed by Dunnett’s test.

The alterations in cremaster arteriolar blood flow after the administration of each flavonoid to rats are illustrated in Figure 9B. The outcomes following the administration of EC and EC following a 24-h incubation period are depicted in Figure 9C. In the EC administration group, a significant increase in arteriolar blood flow was observed 10 min after administration compared to the vehicle administration group, and this change continued for 60 min (Figure 9B). This alteration was not seen when Tax or Q were administered. Conversely, when EC was administered orally in a single dose after a 24-h incubation, the increase in blood flow observed with EC was canceled (Figure 9C).

## 4. Discussion

Flavonoids are found in plants under weakly acidic conditions (e.g., vacuoles) [42], but are exposed to neutral pH conditions in the intestines of mammals. While flavonoids have been observed to be stable in acidic environments, they become more unstable at higher pH levels. A series of tests was therefore conducted to ascertain the stability of three flavonoids at two distinct pH levels.

### 4.1. Differences in Stability of Three Types of Flavonoids

It was observed that under conditions of pH 5, Tax was slightly decomposed, but EC and Q were stable (Figure 1, Figure 2 and Figure 3). Conversely, the residual after 24 or 96 h at pH 7 was 84 or 59% for Tax and 84 or 46% for Q. Nevertheless, a substantial decrease was noted for EC, reaching 76% at 24 h and 33% at 96 h (Figure 1, Figure 2 and Figure 3). Furthermore, a notable browning phenomenon was detected in EC, though this alteration was not observed in Q or Tax (Figure 1, Figure 2 and Figure 3). When Tax and Q were incubated at pH 7, the peaks at *m*/*z* [M-H]^−^ 197 (RT, around 2.0 min), 169 (RT, 2.5 min), 153 (RT, 2.61 min), and 425 (RT, around 8.65) were commonly observed (Figure 2 and Figure 3). As demonstrated by earlier studies on the decomposition processes of Tax [43,44] and Q [45,46,47], these peaks were suggested to be identified as α-oxo-2,4,6-trihydroxybenzeneacetic acid, 2,4,6-trihydroxybenzoic acid, 2,4-dihydroxybenzoic acid, respectively (Figure S3A–C). The compound displaying a mass spectrum of [M-H]− ion at *m*/*z* -425 is hypothesized to be a condensation product in the decomposition process; however, the predicted structure could not be obtained. The presence of a peak with the same mass as the parent compound was detected at RT 8.38 min for taxifolin, suggesting the potential for an isomer [43]. When EC was subjected to incubation at a pH of 7, the composition of its degradation products underwent a temporal change (Figure 1). The peaks commonly observed after 48 and 96 h incubation were [M-H]− ion at *m*/*z*-863 (RT, 1.76 min), 289 (RT, around 4.70 min), 861 (RT, 5.17 min), and 575 (RT, around 9.5 min). The peaks observed only after 48 h incubation were [M-H]− ion at *m*/*z*-577 (RT, 5.71 min) and 863 (RT, 6.81 min). The peaks observed only after 96 h incubation were [M-H]− ion at *m*/*z*-575 (RT, 9.97 min) and 575 (RT, 10.79 min). The presence of a peak with the same mass as the parent compound was detected at RT 4.69 min for EC, suggesting the potential for isomer (+)-catechin [48]. As evidenced by earlier research, the autoxidation of EC also occurs in the absence of enzymes such as polyphenol oxidase and peroxidase [49]. This process depends on the chemical structure of flavanol catechin, resulting in the production of browning substances [50]. It has been suggested that the compound [M-H]− ion at *m*/*z*-575 is a δ-type dehydrodicatechin (δ-DhC2, Figure S3D), the compound showing [M-H]− ion at *m*/*z*-577 is a type of β-type DhC (Figure S3E), [M-H]- 861 is a type of (γ/δ-γ)-type dehydrotricatechin (γ/δ-γ DhC3), and [M-H]- 863 is a type of (δ-β/ε)-type dehydrotricatechin (δ-β/ε DhC3, Figure S3F). The conversion to these brown oligomers, which are stable products, is initiated by two subsequent hydrogen absorptions leading to o-quinone formation with simultaneous reduction of oxygen species. The present study found that the number and size of these condensation product peaks increased after 96 h of incubation compared with the results after 48 h of incubation at pH 7 (Figure 1).

### 4.2. Conformation-Dependent Reactivity of Three Flavonoids with O_2_^•−^

In addition, at a pH of 5, all flavonoids exhibited O_2_^•−^-scavenging activity at any concentration in both incubation periods, whereas at a pH of 7, the reactivity of each compound was observed to differ (Figure 4, Figure 5 and Figure 6). Following a 45-min incubation period, which was designed to simulate the time it takes for the chemicals to reach the intestines, Q exhibited O_2_^•−^ scavenging activity at all concentrations. Conversely, EC and Tax displayed O_2_^•−^ production-promoting activity at low concentrations (Figure 4, Figure 5 and Figure 6). The pro-oxidant activity in Tax reached its peak at approximately 50% at 50 μM, whereas that in EC reached its peak at approximately 65% at 25 μM. This result indicates that EC exhibited higher pro-oxidant activity. It has been reported that at pH 7 or higher, the B-ring phenolic hydroxyl group undergoes auto-oxidation and generates reactive oxygen species (ROS) as shown in Figure S4. Following a 24-hr incubation period, the prooxidant activity in EC disappeared, whereas it was maintained in Tax. These results indicate that Tax is more stable than EC, as shown in Figure 1 and Figure 2, and that the intact form of Tax remains largely even after 24 h of incubation. Previous electrochemical analysis has demonstrated that the initial reversible oxidation of flavonoids is triggered by the presence of two hydroxy substituents at the ortho position [3,4] of the B ring, resulting in the formation of quinones. Additionally, the second irreversible oxidation peak is known to vary significantly depending on the presence of a hydroxyl group at the C3 position. All three of the flavonoids used in the study have this structure. Nevertheless, there has been limited research into the role that C-ring structures play in redox reactions [51].

The present study constitutes a comparison of the redox properties of these three compounds in their interaction with O_2_^•−^, and it was found that clear pH-dependent differences were present. At pH 7, taxifolin, which lacks the C2–C3 double bond of quercetin, and epicatechin, which further lacks the 4-position ketone group, additionally showed significant O_2_^•−^ production-enhancing activity at low concentration. These results suggest that the C2–C3 double bond of flavonoids is a structure that affects O_2_^•−^ production enhancement or elimination.

It was reported that the second peak, which appears more positive than the first peak seen in Q during oxidation, is not seen in Tax in previous electrochemical analyses [52]. It has also been reported that EC produces a characteristic and strong irreversible oxidation peak that is not seen in these two flavonoids, and the results of this study support their electrochemical response. Furthermore, it was established that EC and Tax facilitate the production of O_2_^•−^ at low concentrations while exhibiting a scavenging effect at high concentrations, thereby indicating that the quantitative ratio of EC, Tax, and O_2_^•−^ modulates this bidirectional reaction. To enhance comprehension of the chemical reactivity of these flavonoids, in silico computational analyses were performed (Figure 7). The RMSF values of the B ring of each flavonoid calculated by molecular dynamics showed significantly higher values for EC than for Tax and Q (Figure 7A). These results suggested that EC is the most reactive compound of the test compounds. The reason behind this observation was the following: an extensive conformational change in the B ring of EC was detected between 4600 and 4610 ps, a change that was not observed in Tax or Q (Figure 7B).

After 24 h of incubation, the O_2_^•−^ production-promoting activity of EC disappeared, whereas that of Tax was maintained. These differences are due to the difference in stability between EC and Tax as shown in Figure 1 and Figure 2. It has been observed that some polyphenols undergo oxidation at neutral pH and are regenerated with the O_2_^•−^ produced during the process as a reducing agent [53,54]. At a pH of 7, Tax underwent this regeneration process and was considered to be apparently stable, as demonstrated in Figure 2. Conversely, within the EC, the absence of regeneration was attributed to the production of brown condensation products by oxidation reactions, thereby indicating a decline in intact EC, as shown in Figure 1.

### 4.3. Sympathetic Nervous Activation and the Three-Dimensional Structure of Flavonoids

It was well investigated that the bioavailability of these flavonoids is poor [12,55,56]. Although a few percent of orally ingested flavonoids are absorbed, most of them are metabolized in intestinal epithelial cells and the liver and rarely exist as intact compounds in the blood [57]. Animal studies have shown that repeated administration of flavonoids to rodents can alter the gut microbiota [58]. However, it is unclear whether such changes occur in humans. At least, a single oral administration, as in the present experimental conditions, is unlikely to affect the gut microbiota. The specific bioaccessibility described above complicates the elucidation of the mechanism of beneficial action of flavonoids in humans. For example, it has been well documented that a significant increase in flow-mediated vasodilation (FMD) is observed two hours after ingesting foods rich in flavanol EC [17]. We reported that it is possible to reproduce the significant changes in hemodynamics after flavanol EC administration by measuring the blood flow in the rat cremaster arterioles [24]. Our previous studies have also shown that a single oral intake of flavanol induces significant changes in metabolic pathways, such as browning in adipose tissue and muscle hypertrophy, through increased sympathetic nervous activity [12]. In addition, the administration of flavanol also activates the hypothalamic–pituitary–adrenal (HPA) axis [26], which is a stress response system, making it clear that flavanol is a stressor. Based on the above, in the next stage of this study, we used two types of experimental systems to comparatively analyze the effects of these flavonoids on the sympathetic–adrenal medulla (SAM) axis. The sympathetic–adrenal medulla axis (SAM axis) activated by flavanol is the sympatho–neural system (nervous system) in which NA is released from the sympathetic ganglion to the effector as heart and blood vessels [59], and the sympatho–adrenomedullary system (endocrine system), which involves the secretion of AD into the bloodstream from the adrenal medulla via acetylcholine projections from the sympathetic preganglionic ganglia [60,61]. The former nervous system is known to respond immediately to small environmental changes such as cold, exercise, and active escape behavior. On the other hand, the latter endocrine system is only detected when exposed to severe stress such as immobility, emotional stress, shock, or fear [59]. In this study, we examined the nervous system using changes in blood flow in the rat cremaster artery and the endocrine system using urinary catecholamine excretion in mice as indicators. Among all flavonoids, only EC showed changes in blood flow at 10 μg/kg, and this effect disappeared after 24 h of incubation (Figure 8). Additionally, only EC showed an increase in urinary catecholamine concentration, a hormone, at a dose of 100 μg/kg (Figure 9). The optimal doses of EC in these two experiments differed by a factor of ten, a discrepancy that is explained by the previously mentioned factors.

In this study, we demonstrated that the difference in the C-ring structure of flavonoids significantly affects their stability and redox properties, and that these differences are related to their ability to activate the sympathetic nervous system. For example, EC, which is easily oxidized and unstable, exhibited a significant sympathetic nervous activity enhancement effect, whereas the stable Tax and the antioxidant Q did not show such changes (Figure 9B). Moreover, the enhancement of sympathetic nerve activity observed in EC was not observed in EC after oxidation had progressed and the pro-oxidant activity had disappeared (Figure 9C). This suggests that the bioregulatory effect of flavonoids involves ROS produced by compounds at neutral pH, such as in the digestive tract. It has been reported that the oxidative degradation process of such flavanols is deeply involved in the manifestation of physiological effects. For example, Wu et al. have shown that flavanol epigallocatechin-3-gallate (EGCG) and a fraction containing its autoxidation products were more effective in improving insulin sensitivity in db/db mice at low doses [62]. In previous studies, we also confirmed that ROS derived from flavanols are involved in their effects, as the blood flow-increasing action was cancelled when flavanols, including EC, were co-administered with the ROS scavenger N-acetylcysteine [39]. Conversely, transient receptor potential vanioid1 (TRPV1) and transient receptor potential ankylin 1 (TRPA1), which are predominantly expressed in the enteric nervous system, possess ROS-sensitive domains [12,63]. It has been established that these channels are activated by ROS, and the resulting signals that are transmitted to the central nervous system induce stress response reactions [64]. Moreover, when flavanol was co-administered with a TRP channel inhibitor, the blood flow-increasing effect was negated [39]. This finding suggests the involvement of these channels in the mechanism of action, although further experimentation is required to provide definitive evidence.

### 4.4. Limitations and Perspectives

These results indicate that it is not the effect of flavanol EC oxide itself, but rather the reactive oxygen species (ROS) generated when EC is oxidized under neutral pH conditions similar to those in the intestine that play a crucial role in sympathetic nerve activity. Furthermore, the hypothesis that ROS generated from flavanols are recognized by TRP channels expressed on digestive tract sensory nerves, thereby inducing a stress response and activating the sympathetic nervous system, has gained significant support. To verify this hypothesis, it is essential to measure sympathetic nerve activity using electrochemically stable EC derivatives, and TRP channel knockout mice to clarify the role of ROS generated in the digestive tract. Furthermore, elucidating the relationship between the chemical changes of diverse flavonoids in the digestive tract and the expression of physiological functions is crucial.

## 5. Conclusions

In this experiment, we conducted a comparative analysis of the stability, redox properties, and sympathetic activation ability of three types of flavonoids characterized by divergent C-ring structures. Our findings revealed that flavanols exhibited lower stability, generated ROS under neutral conditions, such as those in the digestive tract, and enhanced sympathetic activity. In contrast, flavanonol, despite producing ROS analogous to those of flavanol, was stable and lacked bioactivity. Flavanol exhibited robust antioxidant properties and did not exhibit sympathetic activation effects. These observations suggest that the C-ring structure of flavonoids plays a critical role in determining their stability and redox properties. The relationship between flavonoid structure and redox properties has been extensively debated. A substantial proportion of this research has elucidated the role of the number of hydroxyl groups on the B ring in determining antioxidant activity [22,65,66]. The present study is original in its focus on the C ring structure, in addition to a comparison of redox properties, and also in its examination of the relationship with physiological effects expressed after ingestion. Furthermore, it has been proposed that the ROS generated by flavanols in the gut may elicit beneficial effects through sympathetic activation. The relationship between the structure of flavonoids, their redox properties, and bioactivity requires further investigation into using in silico, in vitro, and in vivo experiments.

## Data Availability

All data are available in the main text or the Supplementary Materials (three tables and four figures).

## Supplementary Materials

The following supporting information can be downloaded at: https://www.mdpi.com/article/10.3390/antiox15020194/s1.
