# Supplementary material for: C-Ring Structure-Dependent Redox Properties of Flavonoids Regulate the Expression of Bioactivity
Source: Antioxidants (Basel). 2026 Feb 2;15(2):194. doi: 10.3390/antiox15020194 (PMC12938522; doi:10.3390/antiox15020194)
Supplement: Supplementary file 1 [file antioxidants-15-00194-s001.zip › antioxidants-4052300-supplementary.pdf]

Table S1. Parameters of EC

Atom	Mass	Charge	VdW*	Well depth	Repulsive exponent	Dispersion exponent	RMSF (Å)
CT (C 1)	12.0110	-0.0160	1.9080	0.1094	12.0000	6.0000	0.1225
HC (H 2)	1.0080	0.0800	1.4870	0.0157	12.0000	6.0000	-
HC (H 3)	1.0080	0.0800	1.4870	0.0157	12.0000	6.0000	-
CT (C 4)	12.0110	0.2000	1.9080	0.1094	12.0000	6.0000	0.0971
H1 (H 5)	1.0080	0.0800	1.3870	0.0157	12.0000	6.0000	-
c3 (C 6)	12.0110	0.3440	1.9080	0.1094	12.0000	6.0000	0.0953
H1 (H 7)	1.0080	0.0800	1.3870	0.0157	12.0000	6.0000	-
o3 (O 8)	16.0000	-0.3620	1.6837	0.1700	12.0000	6.0000	0.1404
c2 (C 9)	12.0110	0.0820	1.9080	0.0860	12.0000	6.0000	0.1252
c2 (C 10)	12.0110	-0.1500	1.9080	0.0860	12.0000	6.0000	0.1427
HA (H 11)	1.0080	0.1500	1.4590	0.0150	12.0000	6.0000	-
c2 (C 12)	12.0110	0.0820	1.9080	0.0860	12.0000	6.0000	0.1246
c2 (C 13)	12.0110	-0.1500	1.9080	0.0860	12.0000	6.0000	0.1324
HA (H 14)	1.0080	0.1500	1.4590	0.0150	12.0000	6.0000	-
c2 (C 15)	12.0110	0.0820	1.9080	0.0860	12.0000	6.0000	0.1195
c2 (C 16)	12.0110	-0.1440	1.9080	0.0860	12.0000	6.0000	0.1192
oh (O 17)	16.0000	-0.5320	1.7210	0.2104	12.0000	6.0000	0.1697
HO (H 18)	1.0080	0.4500	1.0000	0.0000	12.0000	6.0000	-
oh (O 19)	16.0000	-0.5320	1.7210	0.2104	12.0000	6.0000	0.1207
HO (H 20)	1.0080	0.4500	1.0000	0.0000	12.0000	6.0000	-
c2 (C 21)	12.0110	-0.1440	1.9080	0.0860	12.0000	6.0000	0.1129
c2 (C 22)	12.0110	-0.1500	1.9080	0.0860	12.0000	6.0000	0.1518
HA (H 23)	1.0080	0.1500	1.4590	0.0150	12.0000	6.0000	-
c2 (C 24)	12.0110	0.0820	1.9080	0.0860	12.0000	6.0000	0.1856
c2 (C 25)	12.0110	0.0820	1.9080	0.0860	12.0000	6.0000	0.2398
c2 (C 26)	12.0110	-0.1500	1.9080	0.0860	12.0000	6.0000	0.2215
HA (H 27)	1.0080	0.1500	1.4590	0.0150	12.0000	6.0000	-
c2 (C 28)	12.0110	-0.1500	1.9080	0.0860	12.0000	6.0000	0.1509
HA (H 29)	1.0080	0.1500	1.4590	0.0150	12.0000	6.0000	-
oh (O 30)	16.0000	-0.5320	1.7210	0.2104	12.0000	6.0000	0.4333
HO (H 31)	1.0080	0.4500	1.0000	0.0000	12.0000	6.0000	-
oh (O 32)	16.0000	-0.5320	1.7210	0.2104	12.0000	6.0000	0.2332
HO (H 33)	1.0080	0.4500	1.0000	0.0000	12.0000	6.0000	-
OH (O 34)	16.0000	-0.6800	1.7210	0.2104	12.0000	6.0000	0.1110
HO (H 35)	1.0080	0.4000	1.0000	0.0000	12.0000	6.0000	-

*VdW: van der Waals interaction parameter

Table S2. Parameters of Tax

Atom	Mass	Charge	VdW*	Well depth	Repulsive exponent	Dispersion exponent	RMSF (Å)
c2 (C 1)	12.0110	-0.1500	1.9080	0.0860	12.0000	6.0000	0.1364
HA (H 2)	1.0080	0.1500	1.4590	0.0150	12.0000	6.0000	-
c2 (C 3)	12.0110	-0.1500	1.9080	0.0860	12.0000	6.0000	0.1413
HA (H 4)	1.0080	0.1500	1.4590	0.0150	12.0000	6.0000	-
c2 (C 5)	12.0110	0.0820	1.9080	0.0860	12.0000	6.0000	0.0992
c2 (C 6)	12.0110	0.0820	1.9080	0.0860	12.0000	6.0000	0.1026
c2 (C 7)	12.0110	-0.1500	1.9080	0.0860	12.0000	6.0000	0.1247
HA (H 8)	1.0080	0.1500	1.4590	0.0150	12.0000	6.0000	-
c2 (C 9)	12.0110	-0.1440	1.9080	0.0860	12.0000	6.0000	0.1016
c3 (C 10)	12.0110	0.3440	1.9080	0.1094	12.0000	6.0000	0.1019
H1 (H 11)	1.0080	0.0800	1.3870	0.0157	12.0000	6.0000	-
c3 (C 12)	12.0110	0.2610	1.9080	0.1094	12.0000	6.0000	0.0995
H1 (H 13)	1.0080	0.0800	1.3870	0.0157	12.0000	6.0000	-
c2 (C 14)	12.0110	0.4230	1.9080	0.0860	12.0000	6.0000	0.1050
o= (O 15)	16.0000	-0.5700	1.6612	0.2100	12.0000	6.0000	0.1212
c2 (C 16)	12.0110	0.0860	1.9080	0.0860	12.0000	6.0000	0.1110
c2 (C 17)	12.0110	0.0820	1.9080	0.0860	12.0000	6.0000	0.1052
c2 (C 18)	12.0110	-0.1500	1.9080	0.0860	12.0000	6.0000	0.1111
HA (H 19)	1.0080	0.1500	1.4590	0.0150	12.0000	6.0000	-
c2 (C 20)	12.0110	0.0820	1.9080	0.0860	12.0000	6.0000	0.1136
c2 (C 21)	12.0110	-0.1500	1.9080	0.0860	12.0000	6.0000	0.1301
HA (H 22)	1.0080	0.1500	1.4590	0.0150	12.0000	6.0000	-
c2 (C 23)	12.0110	0.0820	1.9080	0.0860	12.0000	6.0000	0.1222
o3 (O 24)	16.0000	-0.3620	1.6837	0.1700	12.0000	6.0000	0.1460
oh (O 25)	16.0000	-0.5320	1.7210	0.2104	12.0000	6.0000	0.1131
HO (H 26)	1.0080	0.4500	1.0000	0.0000	12.0000	6.0000	-
oh (O 27)	16.0000	-0.5320	1.7210	0.2104	12.0000	6.0000	0.1237
HO (H 28)	1.0080	0.4500	1.0000	0.0000	12.0000	6.0000	-
oh (O 29)	16.0000	-0.6800	1.7210	0.2104	12.0000	6.0000	0.1194
HO (H 30)	1.0080	0.4000	1.0000	0.0000	12.0000	6.0000	-
oh (O 31)	16.0000	-0.5320	1.7210	0.2104	12.0000	6.0000	0.0980
HO (H 32)	1.0080	0.4500	1.0000	0.0000	12.0000	6.0000	-
oh (O 33)	16.0000	-0.5320	1.7210	0.2104	12.0000	6.0000	0.1429
HO (H 34)	1.0080	0.4500	1.0000	0.0000	12.0000	6.0000	-

*VdW: van der Waals interaction parameter

Table S3. Parameters of Q

Atom	Mass	Charge	VdW*	Well depth	Repulsive exponent	Dispersion exponent	RMSF (Å)
c2 (C 1)	12.0110	-0.1500	1.9080	0.0860	12.0000	6.0000	0.1840
HA (H 2)	1.0080	0.1500	1.4590	0.0150	12.0000	6.0000	-
c2 (C 3)	12.0110	-0.1500	1.9080	0.0860	12.0000	6.0000	0.1786
HA (H 4)	1.0080	0.1500	1.4590	0.0150	12.0000	6.0000	-
c2 (C 5)	12.0110	0.0820	1.9080	0.0860	12.0000	6.0000	0.1130
c2 (C 6)	12.0110	0.0820	1.9080	0.0860	12.0000	6.0000	0.1446
c2 (C 7)	12.0110	-0.1500	1.9080	0.0860	12.0000	6.0000	0.2005
HA (H 8)	1.0080	0.1500	1.4590	0.0150	12.0000	6.0000	-
c2 (C 9)	12.0110	0.0280	1.9080	0.0860	12.0000	6.0000	0.1555
c2 (C 10)	12.0110	0.0480	1.9080	0.0860	12.0000	6.0000	0.1519
c2 (C 11)	12.0110	0.0910	1.9080	0.0860	12.0000	6.0000	0.1177
c2 (C 12)	12.0110	0.4690	1.9080	0.0860	12.0000	6.0000	0.1119
o= (O 13)	16.0000	-0.5700	1.6612	0.2100	12.0000	6.0000	0.1088
c2 (C 14)	12.0110	0.0860	1.9080	0.0860	12.0000	6.0000	0.1154
c2 (C 15)	12.0110	0.0820	1.9080	0.0860	12.0000	6.0000	0.1017
c2 (C 16)	12.0110	-0.1500	1.9080	0.0860	12.0000	6.0000	0.1162
HA (H 17)	1.0080	0.1500	1.4590	0.0150	12.0000	6.0000	-
c2 (C 18)	12.0110	0.0820	1.9080	0.0860	12.0000	6.0000	0.1071
c2 (C 19)	12.0110	-0.1500	1.9080	0.0860	12.0000	6.0000	0.1656
HA (H 20)	1.0080	0.1500	1.4590	0.0150	12.0000	6.0000	-
c2 (C 21)	12.0110	0.0820	1.9080	0.0860	12.0000	6.0000	0.1537
o3 (O 22)	16.0000	-0.1590	1.6837	0.1700	12.0000	6.0000	0.2007
oh (O 23)	16.0000	-0.5320	1.7210	0.2104	12.0000	6.0000	0.1022
HO (H 24)	1.0080	0.4500	1.0000	0.0000	12.0000	6.0000	-
oh (O 25)	16.0000	-0.5320	1.7210	0.2104	12.0000	6.0000	0.1108
HO (H 26)	1.0080	0.4500	1.0000	0.0000	12.0000	6.0000	-
oh (O 27)	16.0000	-0.5270	1.7210	0.2104	12.0000	6.0000	0.1435
HO (H 28)	1.0080	0.4500	1.0000	0.0000	12.0000	6.0000	-
oh (O 29)	16.0000	-0.5320	1.7210	0.2104	12.0000	6.0000	0.1868
HO (H 30)	1.0080	0.4500	1.0000	0.0000	12.0000	6.0000	-
oh (O 31)	16.0000	-0.5320	1.7210	0.2104	12.0000	6.0000	0.1256
HO (H 32)	1.0080	0.4500	1.0000	0.0000	12.0000	6.0000	-

*VdW: van der Waals interaction parameter

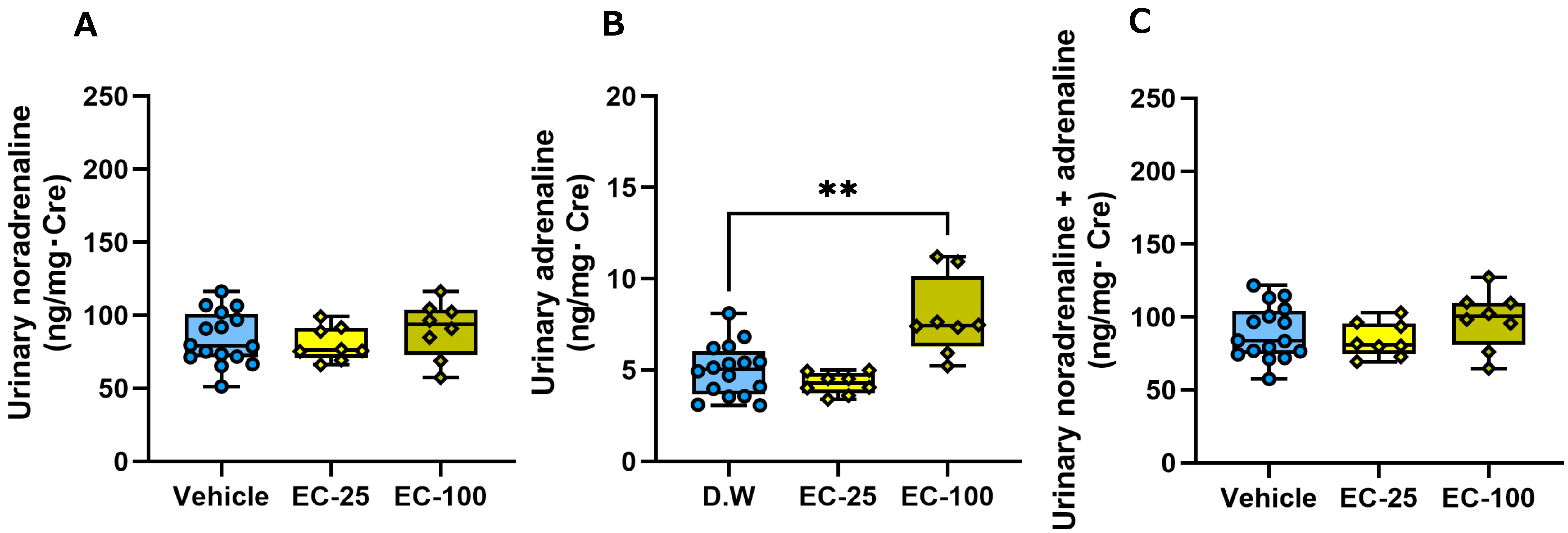

fig.S1 Dose-response study of (-)-epicatechin (25 or 100 µg/kg, urinary excretion of catecholamines). A, adrenaline (AD); B, noradrenalin (NA); C, Total catecholamine (CA). The excretion of CA was expressed as a ratio with the urinary creatinine concentration. Data represents the mean ± SD (n = 8, each), # $p<0.1$, * $p < 0.05$ compared to vehicle, after Kruskal-Wallis test followed by followed by Dunn's tests.

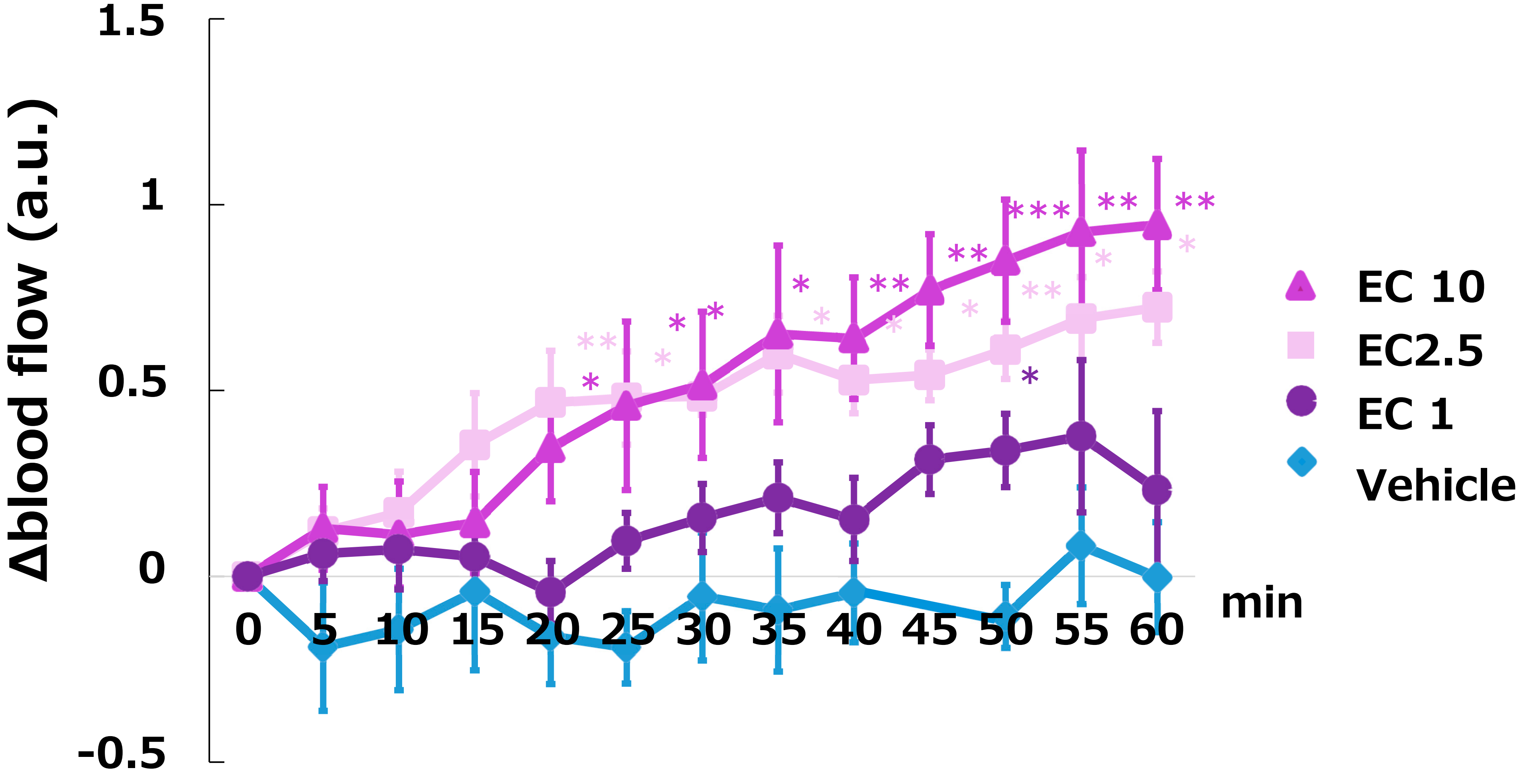

fig. S2 Dose-response study of (-)-epicatechin (1, 2.5 or 10 μg/kg), change of blood flow in rat cremaster arteriole). Data represents the mean ± SD (n = 6, each), , * p < 0.05, ** p < 0.01, compared to vehicle, after two-way ANOVA tests followed by Dunnett's test.

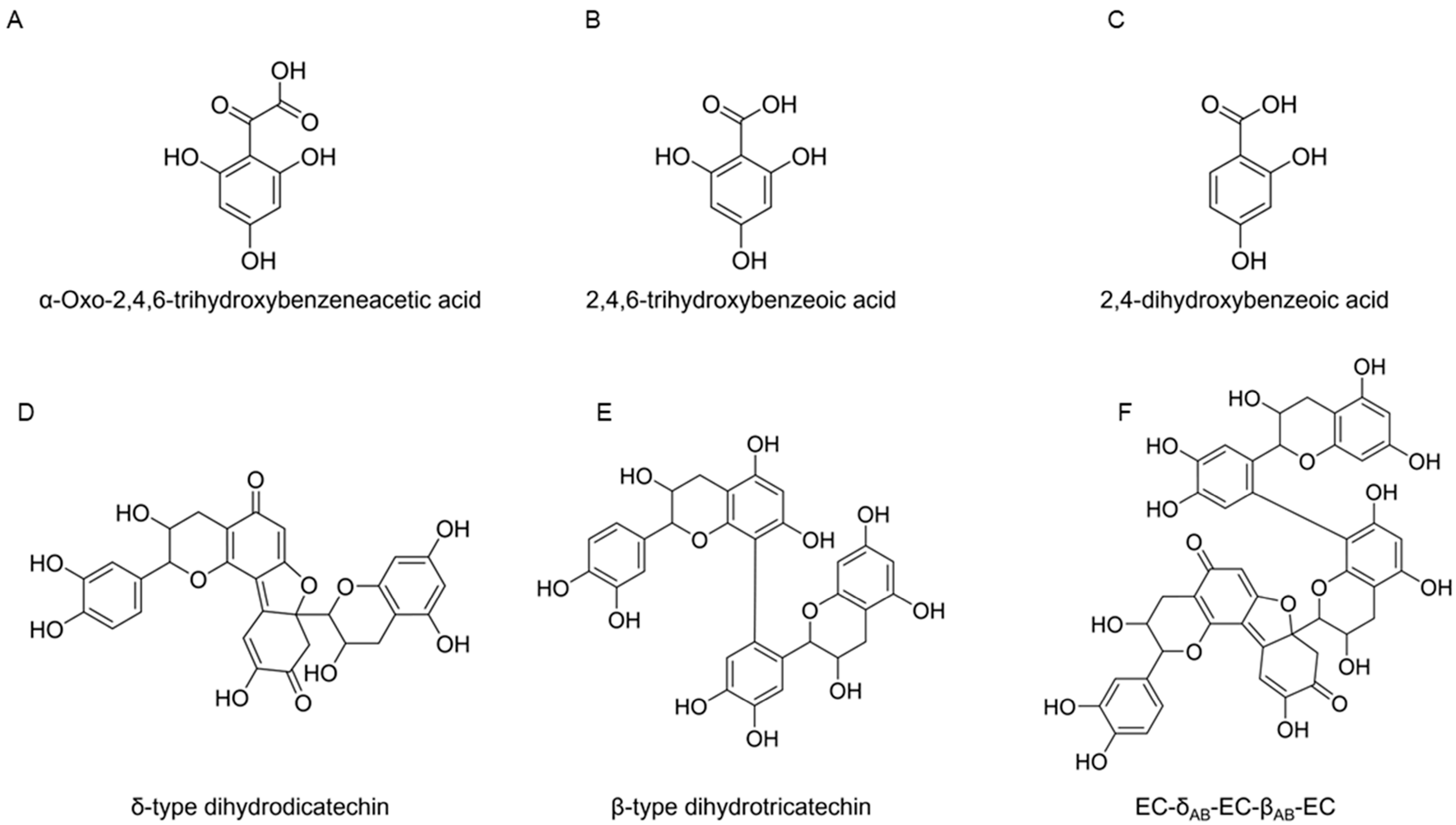

Figure S3 Chemical structure of the peak detected bt LC-MS after incubation of EC, Tax or Q

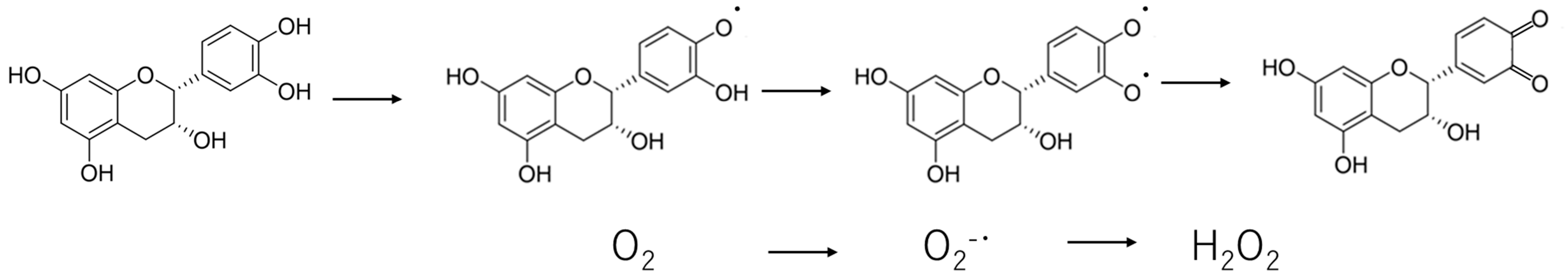

Figure S4 Scheme of epicatechin autooxidation at a pH of >7 coupled with reactive oxygen generation
